# A Novel Microduplication in the Neurodevelopmental Gene *SRGAP3* That Segregates with Psychotic Illness in the Family of a COS Proband

**DOI:** 10.1155/2011/585893

**Published:** 2011-09-12

**Authors:** Nicole K. A. Wilson, Yohan Lee, Robert Long, Karen Hermetz, M. Katharine Rudd, Rachel Miller, Judith L. Rapoport, Anjené M. Addington

**Affiliations:** ^1^Child Psychiatry Branch, National Institute of Mental Health, 10 Center Drive, Building 10/Room 3N202, Bethesda, MD 20892, USA; ^2^Department of Human Genetics, Emory University School of Medicine, 615 Michael Street, Room 315, Atlanta, GA 30322, USA

## Abstract

Schizophrenia is a debilitating mental disorder affecting approximately 1% of the world's population. Childhood onset schizophrenia (COS), defined as onset before age 13, is a rare and severe form of the illness that may have more salient genetic influence. We identified a ~134 kb duplication spanning exons 2–4 of the *Slit-Robo GTPase-activating protein 3* (*SRGAP3*) gene on chromosome 3p25.3 that tracks with psychotic illness in the family of a COS proband. Cloning and sequencing of the duplication junction confirmed that the duplication is tandem, and analysis of the resulting mRNA transcript suggests that the duplication would result in a frame shift mutation. This is the first family report of a *SRGAP3* copy number variant (CNV) in schizophrenia. Considering that *SRGAP3* is important in neural development, we conclude that this *SRGAP3* duplication may be an important factor contributing to the psychotic phenotype in this family.

## 1. Introduction

This paper documents a duplication in the neurodevelopmental gene *Slit-Robo-GTPase activating protein 3* (*SRGAP3*; entrez gene ID: 9901) that segregates with psychotic illness in the family of a patient with childhood onset schizophrenia (COS). Schizophrenia is a severe and debilitating mental disorder characterized by hallucinations, delusions, flat and/or inappropriate affect, and cognitive impairment. It is a multifactorial disease, involving many unknown genetic as well as environmental factors. Although many potential genetic contributors have been identified, penetrance is generally below 20% [1]. Childhood onset schizophrenia, defined as onset before age 13, is a rare and severe form of the illness that may be particularly informative for genetic studies [2–4].

High-density microarrays are a powerful tool for detecting submicroscopic chromosomal duplications and deletions with high resolution and efficiency [5]. COS probands have an increased rate of rare copy number variants (CNVs) and a higher rate of other cytogenetic abnormalities [4]. A novel CNV of particular interest was an ~134 kb duplication within the *SRGAP3* gene on chr3p25.2-3p25.4 that was identified in a COS proband [4]. The duplication was transmitted from the father with schizotypal and avoidant personality disorders to the proband and his brother with schizophrenia. This is the first family report of a *SRGAP3* CNV in schizophrenia and related disorders.

In healthy individuals, *SRGAP3* is highly expressed in fetal and adult brain, and this difference is particularly striking in brain regions involved in higher cognitive function, learning, and memory [6]. *SRGAP3* abnormalities have been observed at the genomic and expression level in several brain disorders including mental retardation [6–8], Parkinson's disease [9], and schizophrenia although previous schizophrenia reports did not have family data [10]. Additionally, the region of chromosome 3 containing *SRGAP3* has been implicated in linkage studies of schizophrenia [11]. Here, we expand on our initial report of this discovery with subsequent cloning and sequencing of the duplication junction to investigate a *SRGAP3* duplication that segregates with psychotic illness in the family of a patient with childhood onset schizophrenia (COS).

## 2. Case Presentation

### 2.1. NSB 499

The proband, NSB499, walked and began speaking within normal limits but was placed in special education (no specific therapies) starting in the first grade primarily because of poor peer relationships dating back to his early childhood years. Starting around age 11, he became confused and disorganized and subsequently started to have auditory and visual hallucinations. He was hospitalized several times beginning around age 13 and his social and academic functioning deteriorated; he described “not being able to feel emotions anymore” and continued to hear voices telling him to hurt himself and others. The proband also experienced visual hallucinations of monster's faces, depressed feelings, anhedonia, sleep and appetite disturbances, and suicidal ideation. At age 14, the proband was admitted to our study and diagnosed with childhood onset schizophrenia, as well as general anxiety disorder, panic disorder, agoraphobia, and depression.

### 2.2. NSB 619

NSB619, the proband's older brother, also inherited the *SRGAP3* duplication from the father. Although he too had normal developmental milestones, he was placed in special education (no specific therapies) in the first grade. NSB 619 experienced his first psychotic episode at age 17, and he was initially diagnosed with schizophrenia at age 18. The patient reported multiple delusions, visual hallucinations, and auditory hallucinations consisting of running commentary between voices conversing with each other. After schizophrenia onset, NSB 619 was incapacitated in his social functioning; he reported having no friends, experiencing discomfort in social situations, and lacking any activities that he enjoyed doing. During interview, it was noted that the patient had poor eye contact and inappropriate facial expressions and did not appear capable of comprehending interview questions.

### 2.3. NSB 617

The father, NSB 617, reported being depressed as a child. He was held back in school twice, placed in special education, and did not graduate until he was 20 years old. Although he was married, at interview he reported having no close friends and experiencing discomfort in social situations. He also reported that he had not worked for years and spent most of his time watching TV. He exhibited impoverished speech, poor hygiene, constricted affect, and magical thinking and often made vague and irrelevant comments during interview. At interview he met criteria for schizotypal personality disorder, avoidant personality disorder, and a history of major depressive disorder.

### 2.4. Noncarrier Family Members

The mother of this family, NSB 618, was diagnosed with schizotypal and avoidant personality disorders as well as a history of major depressive disorder. The sister of the proband, NSB 622, was diagnosed with schizoid personality disorder although she was employed, married with two children, and acted normally in natural social situations. The eldest brother, NSB 621, was diagnosed with paranoid personality disorder. Finally, the second eldest brother, NSB 620, was placed in special education and held back in school twice.

## 3. Results

As part of whole-genome scans using Illumina 1M microarrays, we identified a ~134 kb duplication in exons 2–4 of the *Slit-Robo-GTPase activating protein 3 *(*SRGAP3*) gene that is carried by the father and two schizophrenic brothers described above, but not by the mother or by the other three siblings. To characterize the location of the duplication, we performed fluorescence in situ hybridization (FISH) on metaphase chromosomes prepared from patient-derived lymphoblastoid cell lines. Signals from fosmids corresponding to the duplication were observed at the ends of the short arms of chromosome 3. Signals were not observed on any other chromosomes, consistent with a local duplication (see Supplementary Figure 1 in Supplementary material available online at doi: 10.1155/2011/585893).The ~134 kb duplication was too small to resolve by interphase FISH, thus, we determined the exact location of the duplication using high-resolution array comparative genome hybridization (CGH) and the orientation by using duplication junction cloning and breakpoint sequencing.

**Figure 1:**
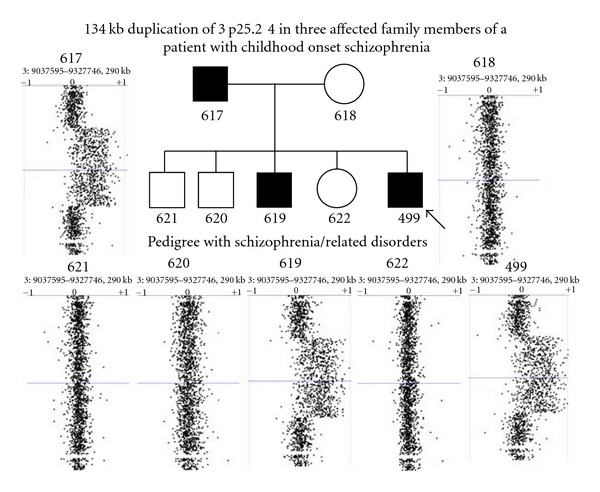
High-resolution microarray analysis of the family of a childhood onset schizophrenia proband. Results revealed a gain on the short arm of chromosome 3 for the proband (NSB499), his father (NSB617), and his brother (NSB619) with the minimum duplication coordinates of chromosome 3: 9,111,177–9,245,155. Arrow on the pedigree indicates the COS proband. *Clinical descriptions*: 617: avoidant PD, schizotypal PD, history of major depressive disorder, held back in school twice. 618: avoidant PD, schizotypal PD, history of major depressive disorder. 621: paranoid PD. 620: held back in school twice. 619: schizophrenia. 622: schizoid PD. 499: childhood onset schizophrenia.

Microarray analysis revealed a gain of the short arm of chromosome 3 for the father and two sons from the same family. The minimum duplication coordinates in these three family members were chr3: 9,111,177–9,245,155 (Build 37, hg19) (Figure 1).

To determine the orientation of the duplication, we cloned the junction using a PCR strategy designed to capture either tandem or inverted duplications [12]. Sequencing the breakpoint junction PCR product revealed a ~134 kb tandem duplication with the following coordinates: chr3: 9,111,132–9,245,356. We aligned the sequence of the tandem duplication breakpoint, which revealed 2 basepairs of microhomology at the junction (Supplementary Figure 2).

**Figure 2:**
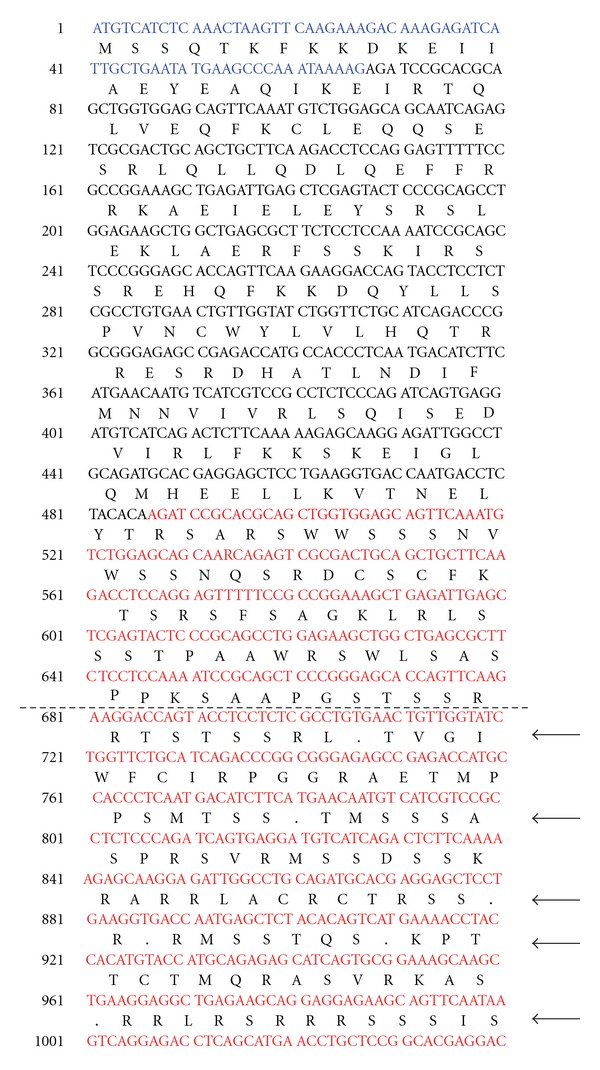
Predicted mRNA sequence and corresponding amino acid sequence generated using Sequencher 3.0. Exon 1 is shown in blue, and the tandem duplication of exons 2–4 is shown in pink. The duplication causes a frameshift which leads to six stop codons in the first 185 amino acids after the start of the duplication (represented by dots; see arrows). These data suggest that the transcript would not be functional.

Sequencher analysis of the NM_014850.2 transcript of *SRGAP3* revealed that the tandem duplication would result in a shift of the mRNA reading frame, ultimately resulting in six stop codons in the first 185 amino acids after the start of the duplication, with the first stop codon falling after the 73rd amino acid in the duplicated portion (Figure 2).

## 4. Discussion

This paper documents the first duplication in the neurodevelopmental gene *SRGAP3* that segregates with psychotic illness in the family of a patient with childhood onset schizophrenia. This tandem duplication results in mRNA that undergoes a shift of the reading frame that would ultimately encode a stop codon, rendering this functionally a null mutation (Figure 2).

Schizophrenia is a multifactorial disease with multiple unknown genetic and environmental factors [13]. It is unlikely that any given case will be caused by a single mutation or that the phenotype resulting from any given mutation will be consistent [1, 13]. One example of this is the widely studied schizophrenia susceptibility gene *DISC1* that was first identified as a balanced translocation in a large Scottish family [14]. Of 77 family members studied, 34 carried the genetic abnormality [14]. Of the 34 carriers of the *DISC1* genetic abnormality, 16 had various psychiatric diagnoses ranging from schizophrenia to alcoholism; there were also 5 of the 43 noncarrier family members who had a psychiatric diagnosis [14]. As with the pattern of penetrance observed in the family with a *DISC1* translocation, we would not expect the *SRGAP3* duplication to track perfectly with the psychotic phenotype. This *SRGAP3* duplication is probably only one of many factors contributing to the psychotic phenotype in this family.

**Figure 3:**
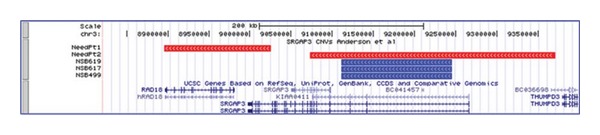
*SRGAP3* duplications reported for a childhood onset schizophrenia proband (NSB499), his brother with schizophrenia (NSB617), and his father with other psychotic features (NSB619)(in blue, ascending order from bottom) aligned with *SRGAP3* duplications previously seen in two adult patients [10] (shown in red).

*SRGAP3* abnormalities have been previously reported in several neurodevelopmental disorders [6–10]. Although noted without comment in a previous genome-wide assessment of CNVs in 1073 cases of schizophrenia and 1148 controls, two patients were identified with duplications in *SRGAP3*, and none of the controls had CNVs in this region although this did not reach significance and no family data was collected [10]. One of these duplications encompassed the same *SRGAP3* exons that are duplicated in the family of NSB499 (Figure 3). Abnormalities in *SRGAP3* have also been implicated in intellectual deficiency and Parkinson's disease [6–9]. 

A closer look at the molecular interactions of the *SRGAP3* protein product reveals its potential relevance to neuronal development. The *SRGAP3* protein product is known to enhance intrinsic activity of certain GTPases, particularly Rac1 and to a lesser extent CDC42 [6]. Interestingly, *DISC1* has also been shown to regulate Rac1 activity, and constitutive Rac1 activation results in a decrease in the size of synaptic spines [15]. *SRGAP3* may also regulate the size and density of dendritic spines by way of an inverse F-BAR domain that is characteristic of proteins from the SRGAP family [16]. This domain likely functions by utilizing a convex lipid-binding surface that facilitates the development of synaptic spines [16]. Furthermore, loss of *SRGAP3* results in a decrease in the density of dendritic spines both *in vivo* and *in vitro* [16].

Taken together, our findings and the studies cited above suggest that *SRGAP3 *may play an important role in neural development and that disruption or alteration of this gene may be partially etiologic for several mental disorders. The present study indicates that *SRGAP3* may be a susceptibility gene for COS. Further investigation of association between *SRGAP3* and schizophrenia is warranted.

## Supplementary Material

In the supplementary materials section we include additional descriptions of the materials and methods, two supplementary figures, and a UCSC Genome Browser compatible BED file containing the precise coordinates of the CNV tracks. In the materials and methods section, we describe details for the spotted microarray analysis, FISH analysis, duplication junction cloning, sequencing of the resulting PCR product, and additional analyses of the resulting transcript. Supplemental Figure 1 includes images generated from the metaphase FISH assay and Supplemental Figure 2 includes the genomic sequence read at the duplication junction. Supplemental Figure 3 includes the CNV coordinates formatted for compatible visualization in the UCSC Genome Browser which was used to produce Figure 3 in the manuscript. Finally, we have included information on the grant used to fund this research.
