# Supplementary material for: A Novel Microduplication in the Neurodevelopmental Gene SRGAP3 That Segregates with Psychotic Illness in the Family of a COS Proband
Source: Case Rep Genet. 2011 Sep 12;2011:585893. doi: 10.1155/2011/585893 (PMC3447216; doi:10.1155/2011/585893)
Supplement: Supplementary file 1 — In the supplementary materials section we include additional descriptions of the materials and methods, two supplementary figures, and a UCSC Genome Browser compatible BED file containing the precise coordinates of the CNV tracks. In the materials and methods section, we describe details for the spotted microarray analysis, FISH analysis, duplication junction cloning, sequencing of the resulting PCR product, and additional analyses of the resulting transcript. Supplemental Figure 1 includes images generated from the metaphase FISH assay and Supplemental Figure 2 includes the genomic sequence read at the duplication junction. Supplemental Figure 3 includes the CNV coordinates formatted for compatible visualization in the UCSC Genome Browser which was used to produce Figure 3 in the manuscript. Finally, we have included information on the grant used to fund this research. [file 585893.f1.pdf]

Supplementary Materials: A Novel Microduplication in the Neurodevelopmental Gene *SRGAP3* that Segregates With Psychotic Illness in the Family of a COS Proband

MATERIALS AND METHODS

Patient samples

Patient samples were collected as previously described [1]. The NIMH Institutional Review Board approved sample collection after written and informed consent was obtained from all study participants.

High density genotyping CNV analysis

The Illumina microarray analysis was carried out as previously described [2].

FISH analysis

Metaphase chromosomes from lymphoblastoid cell lines were prepared using standard methods. To determine the location of the duplicated material, we performed FISH using two fosmid probes from the duplicated region, G248P86704D9 and G248P8810B10. We hybridized fosmid probes to metaphase chromosomes and analyzed FISH signals in 10 metaphase spreads per cell line. In all cases, two overlapping fosmid signals were present on the two chromosomes 3, with no signals on other chromosomes. We identified the chromosomes 3 using inverted DAPI images. Although the small size of the duplication precluded interphase analysis of the duplication, our FISH experiments demonstrated that the duplicated sequence was not present on another chromosome, consistent with a local duplication.

High resolution targeted oligonucleotide array CGH analysis

We designed custom oligonucleotide arrays using the 8x15K platform, AMADID # 029459 (Agilent Technologies, Santa Clara, CA). The duplication region was targeted with probes from coordinates chr3: 8,000,000 - 10,000,000 (Build 37, hg19) with a mean probe spacing of 1 probe per 181 bp. We also added a backbone of probes outside of the duplication from coordinates 1-8,000,000 and 10,000,000 - 18,000,000 with a mean probe spacing of 1 probe per 200 kb. Array CGH was performed following the manufacturer's protocol. Genomic DNA was extracted from lymphoblastoid cell lines derived from family members and a control (GM10851, Coriell Cell Repositories, Camden, New Jersey). Patient and control DNA samples were cohybridized in seven separate hybridizations. Arrays were scanned using a GenePix® 4000B scanner (Molecular Devices, Sunnyvale, CA, USA) and signal intensities were evaluated using Feature Extraction Version 9.5.1.1 software (Agilent Technologies, Santa Clara, CA). We used DNA Analytics Version 4.0 software (Agilent Technologies, Santa Clara, CA) to analyze the array data and narrow the duplication breakpoints.

PCR and sequencing

Using the duplication breakpoints sized by array CGH, we designed PCR primers that would amplify the duplication junction either in tandem or inverted orientation [3]. The primer sequences are as follows: NIH_P1.5: GCTGTCAACTTCCAGATTCTGTTTG and NIH_P2.4: GAGCAGCAGCCAAATTAGCTCG. The PCR amplified a 900-bp product in DNA from cell lines derived from NSB499, NSB617, and NSB619, but not other family members. We TA-cloned the PCR product and sequenced 8 individual clones. All inserts had the same sequence and were consistent with a tandem duplication junction. Purified plasmid DNA and live culture stabs are available upon request.

mRNA transcript analysis

To predict the reading frame of the mRNA resulting from the tandem duplication, we used Sequencher® software version 3.0 to analyze the NM_014850.2 transcript of SRGAP3.

Sanger Sequencing

Sequencing was carried out as previously described [4].

Materials and Methods References

1. Addington, A.M. and J.L. Rapoport, *The genetics of childhood-onset schizophrenia: when madness strikes the prepubescent.* Curr Psychiatry Rep, 2009. **11**(2): p. 156-61.
2. Walsh, T., et al., *Rare structural variants disrupt multiple genes in neurodevelopmental pathways in schizophrenia.* Science, 2008. **320**(5875): p. 539-43.
3. Arlt, M.F., et al., *Replication stress induces genome-wide copy number changes in human cells that resemble polymorphic and pathogenic variants.* Am J Hum Genet, 2009. **84**(3): p. 339-50.
4. Hamdan, F.F., et al., *No association between SRGAP3/MEGAP haploinsufficiency and mental retardation.* Arch Neurol, 2009. **66**(5): p. 675-6.

FUNDING

This project was funded by the NIMH DIRP Child Psychiatry Branch Protocol, grant reference #MH002581-20 CPB Childhood Onset Schizophrenia.

Supplemental Figures

Supplemental Figure 1

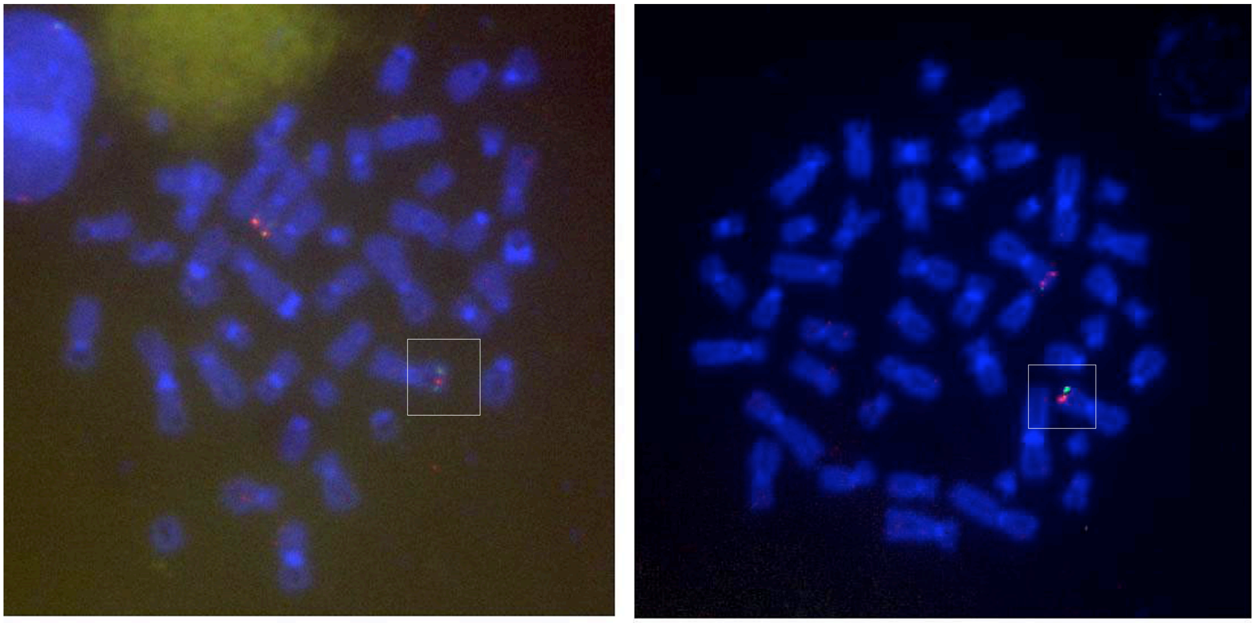

Carrier NSB 619 Noncarrier NSB 618

Supplemental Figure 1: Metaphase FISH signals of a carrier and noncarrier alike were displayed at the end of chromosome 3 and not on any other chromosomes, consistent with a local duplication.

Supplemental Figure 2

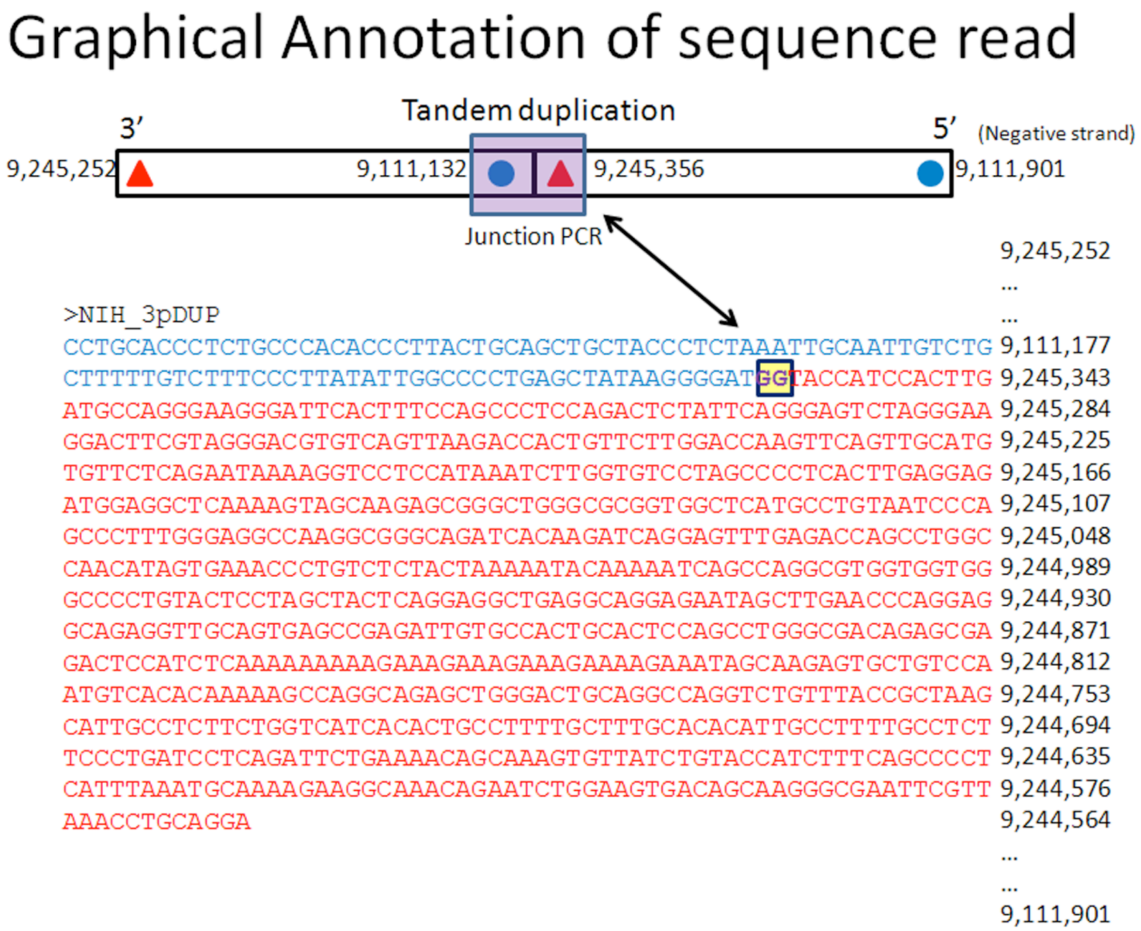


```
                                                              9,245,252
                                                              ...
>NIH_3pDUP                                                    ...
CCTGCACCCTCTGCCCACACCCTTACTGCAGCTGCTACCCTCTAAATTGCAATTGTCTG 9,111,177
CTTTTTGTCTTTCCCTTATATTGGCCCCTGAGCTATAAGGGGATGGTACCATCCACTTG 9,245,343
ATGCCAGGGAAGGGATTCACTTTCCAGCCCTCCAGACTCTATTCAGGGAGTCTAGGGAA 9,245,284
GGACTTCGTAGGGACGTGTCAGTTAAGACCACTGTTCTTGGACCAAGTTCAGTTGCATG 9,245,225
TGTTCTCAGAATAAAAGGTCCTCCATAAATCTTGGTGTCCTAGCCCCTCACTTGAGGAG 9,245,166
ATGGAGGCTCAAAAGTAGCAAGAGCGGGCTGGGCGCGGTGGCTCATGCCTGTAATCCCA 9,245,107
GCCCTTTGGGAGGCCAAGGCGGGCAGATCACAAGATCAGGAGTTTGAGACCAGCCTGGC 9,245,048
CAACATAGTGAAACCCTGTCTCTACTAAAAATACAAAAATCAGCCAGGCGTGGTGGTGG 9,244,989
GCCCCTGTACTCCTAGCTACTCAGGAGGCTGAGGCAGGAGAATAGCTTGAACCCAGGAG 9,244,930
GCAGAGGTTGCAGTGAGCCGAGATTGTGCCACTGCACTCCAGCCTGGGCGACAGAGCGA 9,244,871
GACTCCATCTCAAAAAAAAAGAAAGAAAGAAAGAAAAGAAATAGCAAGAGTGCTGTCCA 9,244,812
ATGTCACACAAAAAGCCAGGCAGAGCTGGGACTGCAGGCCAGGTCTGTTTACCGCTAAG 9,244,753
CATTGCCTCTTCTGGTCATCACACTGCCTTTTGCTTTGCACACATTGCCTTTTGCCTCT 9,244,694
TCCCTGATCCTCAGATTCTGAAAACAGCAAAGTGTTATCTGTACCATCTTTCAGCCCCT 9,244,635
CATTTAAATGCAAAAGAAGGCAAACAGAATCTGGAAGTGACAGCAAGGGCGAATTCGTT 9,244,576
AAACCTGCAGGA                                                9,244,564
                                                            ...
                                                            ...
                                                            9,111,901
```

Supplemental Figure 2: Nucleotide sequence at the tandem duplication breakpoint.

Supplemental Figure 3

browser position chr3:8,821,666-9,400,000
track name=coords description="SRGAP3 CNVs Anderson et al" visibility=2, itemRgb=on

chr3	9111132	9245356	NSB499	500	-	9111132	9245356	0,0,255
chr3	9111132	9245356	NSB617	500	-	9111132	9245356	0,0,255
chr3	9111132	9245356	NSB619	500	-	9111132	9245356	0,0,255
chr3	8895281	9025800	NeedPt1	500	-	8895281	9025800	255,0,0
chr3	9073423	9371030	NeedPt2	500	-	9073423	9371030	255,0,0

Supplemental Figure 3: Text file in BED format uploaded into UCSC genome browser custom tracks to produce Figure 3.
